# Engineered exosomes as drug and RNA co-delivery system: new hope for enhanced therapeutics?

**DOI:** 10.3389/fbioe.2023.1254356

**Published:** 2023-09-26

**Authors:** Haorong Chen, Hanbo Yao, Jiaxin Chi, Chaowei Li, Yilin Liu, Jiayi Yang, Jiaqi Yu, Jiajun Wang, Yongdui Ruan, Jiang Pi, Jun-Fa Xu

**Affiliations:** ^1^ Guangdong Provincial Key Laboratory of Medical Molecular Diagnostics, The First Dongguan Affiliated Hospital, Guangdong Medical University, Dongguan, Guangdong, China; ^2^ Institute of Laboratory Medicine, School of Medical Technology, Guangdong Medical University, Dongguan, Guangdong, China; ^3^ The Marine Biomedical Research Institute, Guangdong Medical University, Zhanjiang, Guangdong, China

**Keywords:** engineered exosomese, drug, RNA, co-delivery system, combination therapies

## Abstract

Chemotherapy often faces some obstacles such as low targeting effects and drug resistance, which introduce the low therapeutic efficiency and strong side effects. Recent advances in nanotechnology allows the use of novel nanosystems for targeted drug delivery, although the chemically synthesized nanomaterials always show unexpected low biocompability. The emergence of exosome research has offered a better understanding of disease treatment and created novel opportunities for developing effective drug delivery systems with high biocompability. Moreover, RNA interference has emerged as a promising strategy for disease treatments by selectively knocking down or over-expressing specific genes, which allows new possibilities to directly control cell signaling events or drug resistance. Recently, more and more interests have been paid to develop optimal delivery nanosystems with high efficiency and high biocompability for drug and functional RNA co-delivery to achieve enhanced chemotherapy. In light of the challenges for developing drug and RNA co-delivery system, exosomes have been found to show very attractive prospects. This review aims to explore current technologies and challenges in the use of exosomes as drug and RNA co-delivery system with a focus on the emerging trends and issues associated with their further applications, which may contribute to the accelerated developments of exosome-based theraputics.

## Introduction

Exosomes are small membrane vesicles (30–150 nm) containing complex lipids, RNAs and proteins, which can be release by prokaryotes, eukaryotes, various cells, and mediate cell to cell communication in physiological and pathological conditions (Lai et al., 2022). As a subset of EV, exosomes were initially recognized as a mechanism for cells to excrete unwanted cell metabolites or other substances. However, in 1983, exosomes were firstly found in reticulocytes of sheep, and Johnstone named them exosomes in 1987 (Johnstone et al., 1987). There are three processes, including the formation of endocytic vesicles, generation of multivesicular bodies (MVBs), and release of exosomes, collectively comprise the biogenesis and development of exosomes (Han et al., 2022). Once exosomes are released from the cell, they can fuse with the plasma membrane of the recipient cell and delivers contents to the intracellular compartment, including proteins, nucleic acid, lipids and metabolites,etc., which play a crucial role in biological function (Dai et al., 2020). Exosomes thus can affect a wide range of physiological processes, such as stem cell maintenance (Batsali et al., 2020), lipid metabolism (Wang et al., 2020), aging speed (Zhang et al., 2017) immune response (Zhang and Yu, 2019), reproductive development (Esfandyari et al., 2021), cardiovascular disease (Zheng et al., 2020), infectious diseases (Schorey et al., 2015), neurodegenerative disease (Guo et al., 2021) and cancer (Paskeh et al., 2022).

RNA therapy refers to the use of nucleic acids-based molecules, which can manipulate gene expression or produce therapeutic proteins for disease treatment. The targets mainly include small interference RNA (siRNA), microRNA (miRNA), antisense oligonucleotides (ASO), and messenger RNA (mRNA) (Gupta et al., 2021). Relative to the nucleic acid therapeutics described above, although the aptamers, sarnas, and CRISPR/CAS, have been less frequently reported, they still hold great potential in the future (Yoon and Rossi, 2018; Zhu and Chen, 2018; Guo et al., 2022). Nucleic acid-based therapies have emerged as promising treatment modalities for a wide variety of diseases, these therapies allow for targeted modulation of gene expression, facilitating the treatment of both genetic and acquired conditions. Nucleic acid-based therapies have shown immense potential in providing innovative treatments for disorders such as cancer (Meng et al., 2022), infectious diseases (Xiao et al., 2019), and autoimmune diseases (Leung et al., 2010). However, for further use of nucleic acid as novel drugs for therapy, controlling pharmacokinetics (PK) parameters of small molecule drugs is still the core challenge, while there are also some remaining issue like how to improve the structural stability, reduce immunogenicity and limit the side effects of RNA molecules (Winkle et al., 2021).

Since small molecule drugs can rapidly diffuse through biological fluids, many biological barriers, and cell membranes which enable small molecules to navigate complex vascular systems and interact with virtually all tissues and cell types in the body. But situations arise such as the difficulty of the drug to breach the inevitable physiological barriers, the occurrence of adverse reactions in the blood or peripheral tissues or the loss of bioavailability or the inability to dissolve easily in the gastrointestinal tract (Zhong et al., 2021a).

Drug delivery systems (DDS) refer to the technological systems that comprehensively regulate the distribution of drugs or biomolecules in living organisms in space, time, and dose (Mainardes and Silva, 2004). Over the past two to three decades, investigators have developed viral and nonviral vectors. However, clinical success is very limited and hampered by challenges that there are no suitable delivery systems. The use of cell secretory nanovesicles, such as exosomes as vehicles for delivery application has created tremendous excitement in the field of gene therapy, likely due to their good pharmacokinetics, biocompatibility, minimal or no inherent toxicity, long plasma half-life, and intrinsic ability to cross biological barriers (Duan et al., 2021; Rezaie et al., 2022). On the one hand, single RNA therapy can effectively promote or inhibit gene expression, which has broad application prospects; On the other hand, single small-molecule drug therapy is still widely exists in clinical practice in anti-tumor drugs and other diseases. Reasonable combination chemotherapy and gene therapy and selection of exosomes as carrier lecturer are the latest idol of disease treatment development (Kobayashi et al., 2020; Zhao et al., 2020).

In this review, we describe how exosomes are produced from donor cells and absorbed by recipient cells, as well as the extraction and purification methods of exosome. Moreover, we emphatically describe the occurrence and development of exosomes as delivery systems for co-delivery of RNAs and chemotherapy drugs, as well as their potential application and current challenges for clinical therapeutic. We believe that this review would enhance our understanding of potential for exosomes as co-delivery systems of RNAs and chemotherapy drugs for novel therapeutic development, and finally promote the development of exosomes for clinical uses.

### Exosomes: biogenesis, secretion, and cellular uptake

Exosomes have been regarded as descendants of the parental cell. Extracellular vesicles (EVs) are usually divided into three groups according to their size and biogenesis: exosomes (50–150 nm), microvesicles (MVs) (100–1,000 nm) and apoptotic bodies (>1,000 nm) (Jin et al., 2022). Exosomes are considered to be of endocytic origin, MVs are produced by budding and blebbing from the plasma membrane and apoptotic bodies are released by cells undergoing programmed cell death and signal cell engulfment (Colombo et al., 2014). Here, we focus on introducing the biogenesis of exosomes, how they are secreted from parent cells and how to be absorbed by recipient cells.

Understanding the biogenesis and secretion of exosomes is particularly important for the further clinical uses of exosomes. So far, multiple kinds of mechanisms involved in exosome biogenesis have been identified.

The Endosomal sorting complex required for transport (ESCRT) machinery is crucial in the biogenesis of exosomes. Defects in ESCRT machinery lead to impaired exosome secretion (Tamai et al., 2010; Tamai et al., 2012), and the development of various pathological conditions (Wei et al., 2015; Han et al., 2019). Tetraspanins CD9, CD63, and CD81 are particularly enriched in the exosomal membrane (Jeppesen et al., 2019). CD63 is abundant on late endosomes, participates in ESCRT-independent sorting of the PMEL luminal domain, and Iron loading leads to secretion of CD63 positive extracellular vesicles (Pols and Klumperman, 2009; van Niel et al., 2011; Yanatori et al., 2021). Moreover, complex lipids such as ceramide can self-associate, forming raft-like structures and contribute to the initial membrane curvature for inward budding to form ILVs (Trajkovic et al., 2008). Different Rab proteins, such as Rab5 (Gorji-Bahri et al., 2021), Rab7 (Sun et al., 2020), Rab27a (Ostrowski et al., 2010), Rab35 (Sato et al., 2008), etc. play important roles in specific steps of exosome biogenesis and secretion. Various N-ethylmaleimide-sensitive fusion attachment protein (SNAP) receptors (SNAREs) families like VAMP2/3/4/7/8 proteins are present in Ilvs (Steegmaier et al., 1999; Chen and Scheller, 2001; Pryor et al., 2004; Marshall et al., 2015; Gordon et al., 2017). Autophagy, regulated by autophagy-related genes such as Atg5 and Atg12-Atg3, determines the outcome of late endosomes and leads to late endosomal acidification (Murrow et al., 2015; Guo et al., 2017). The microenvironment surrounding the cells likewise influences exosome secretion, and can be regulated by physical signals, starvation or hyperglycemia, acidity, hypoxia, liposomes, or even circadian rhythm (Cheng et al., 2019; Debbi et al., 2022; Yeung et al., 2022). Recently, a molecular pathway linking the rigid extracellular matrix to oncogenic signaling and exosome trafficking was proposed, ultimately facilitating tumor progression (Wu et al., 2023). Exosome secretion is a complex and responsive biological process. Inhibition of a single pathway may not completely stop its secretion. Therefore, a multi-targeted approach is required to understand and manipulate exosome secretion, which involves identifying and evaluating all molecular players and pathways involved.

Exosome research has gained traction due to the observed phenotypes resulting from their entry into recipient cells, and their potential as engineered delivery vehicles in therapeutic applications. However, there is still a lack of consensus on the mechanism by which recipient cells uptake exosomes. Endocytosis is well accepted: Exosomes can be internalised by a clathrin-mediated, lipid-raft mediated, caveolin-mediated endocytosis, phagocytosis or micropinocytosis and macropinocytosis (Mulcahy et al., 2014; Gurung et al., 2021). Exosomes release their contents into recipient cells another possible entry mechanism is the direct fusion of their membranes with the cytoplasmic membrane, a process that is engaged by proteins such as SNAREs and the Rab family (Jahn and Sudhof, 1999).

At the same time, the direct contact between the exosome surface and the receptor on the receptor cell was also reported. For instance, Dendritic cell-derived exosomes can directly stimulate T cells due to carrying major histocompatibility complex (MHC) and T cell costimulatory molecules (Schorey et al., 2015). Integrins, lectins/proteoglycans and T cell immunoglobulin and mucin domain-containing protein 4, which also played an integral role in mediating exosome binding to receptor ligands of the cell (Mathieu et al., 2019). The fate of exosomes entering recipient cells is a critical aspect of pharmacokinetics. However, the ultimate destination of exosomal cargo (including potential re-secretion or degradation) remains unassessed.

### Functions and biomedical applications of exsomes

Exosomes are being increasingly utilized in biomedical applications for disease prevention, diagnosis, and treatment, with rapid advancements being made towards their clinical use. In the following passages, we will briefly discuss the potential of exosomes in these areas.

#### Potential biomarker for disease diagnosis

The discovery, validation, and implementation of new biomarkers play a crucial role in improving clinical prognosis (He et al., 2018; Poller et al., 2018). Liquid biopsy offers several advantages, including minimal invasiveness, rapidity, and ease of dynamic monitoring (Lone et al., 2022). Taking the advantages of some properties beyond other sample sources, exosomes have emerged as a noteworthy niche in the field of liquid biopsy, attracting significant attention. While there are ongoing discussions surrounding the purity and cost of exosome extraction, they offer distinct advantages over circulating tumor cells (CTCs) and cell-free tumor DNA (ctDNA). Firstly, exosomes allow for the non-invasive collection of samples from various bodily fluids. They are relatively stable and carry a wide range of molecules, including proteins, nucleic acids, and lipids, which provide valuable insights into the tumor microenvironment. Currently, there is ongoing research exploring the potential of combining exosomal proteins, lipids, RNA, and miRNA in cancer diagnosis and prognostic assessment. In recent studies, a novel multiplex *in situ* detection method has been developed for exosomal miRNA and protein analysis, enabling the quantitative analysis of disease-specific miRNA and surface proteins derived from prostate cancer cells within exosomes in a single reaction (Cho et al., 2019). Exosomes can be isolated from multiple body fluids such as blood, urine, and saliva, facilitating biomarker analysis (Zhang et al., 2018). However, it is important to note that the methods for exosome isolation are still evolving, and variations in protocols may impact the quality and quantity of the obtained exosomes. Additionally, the heterogeneity of exosomes in terms of size, content, and origin poses challenges for standardization and result interpretation (Thery et al., 2018). In contrast, CTCs and ctDNA can also be isolated from peripheral blood, offering non-invasive sample collection and real-time monitoring of tumor progression and treatment response, whcih has been associated with poor prognosis in various cancer types (Yu et al., 2013; Bettegowda et al., 2014). However, CTCs exhibit molecular heterogeneity compared to primary tumor cells, which may affect their representation of the overall tumor biology. Moreover, the lack of standardized protocols for CTC isolation, detection, and characterization limits their widespread clinical application (Lin et al., 2021). Similar to CTC, the low quantities of ctDNA compared to normal cell-free DNA make its detection and quantification challenging, particularly in early-stage cancers (Yu et al., 2021). The sensitive techniques required for ctDNA detection and characterization, such as next-generation sequencing or digital PCR, restrict its accessibility in some clinical settings. Additionally, the genetic alterations detected in ctDNA may not fully capture the tumor heterogeneity, as ctDNA shedding can vary among tumor subclones (Pessoa et al., 2020).

While exosomes encapsulation therapy as a targeted drug delivery technology is still in its early stages of development, advancements in detection methods have made it possible to rapidly analyze and test specific cargo within extracellular vesicles for diagnostic purposes. This feasibility has played a crucial role in promoting the development of extracellular vesicle biomarkers. The pathological transport of exosomes throughout the course of disease development and progression holds potential for disease diagnosis, prognosis, and treatment strategie (Zhou et al., 2020; Yu et al., 2022). In addition to exosomal miRNAs, which have been extensively studied and associated with significant differences in expression profiles between disease and healthy groups, there is a vast array of other cargo present in exosomes. Although specific AUC (area under the curve) values are not provided in this table (Table 1), numerous studies have reported significant differences in exosomal miRNA expression profiles, further underscoring the potential utility of exosomes as biomarkers.

**TABLE 1:** Exosomal contents as biomarkers.

	Disease	Exosome isolation	Sample	Marker	AUC	Tendency	Reference	Remark
Respiratory system	TB	Total exosome isolation reagent (Invitrogen, United States)	Serum; 25 TB patients and 25 HCs	miR-484	0.725	Upregulated	Alipoor et al. (2019)	Combination of mir-484 mir-425 and mir-96 (AUC:0.78)
				miR-425	0.669	Upregulated		
				miR-96	0.628	Upregulated		
		ExoQuick Kit (System Biosciences, United States)	Plasma; 44 PTB patients, 22 TBM patients and 39 HCs	Combination of mircroRNAs(miR-20a, 20b, 26a, 106a, 191, and 486)	0.81/0.85	All upregulated	Hu et al. (2019)	Distinguish PTB/TBM patients from HCs(AUC:0.81/0.85)
Nervous system	AD	ExoQuick exosome precipitation solution (System Biosciences, CA)	Plasma; 73 AD patients, 71 AMCI patients, and 72 HCs	Ab42	0.93	Upregulated	Jia et al. (2019)	Distinguish AMCI patients from HCs(AUC:0.74/0.79/0.83)
				T-tau	0.89	Upregulated		
				P-T181-tau	0.88	Upregulated		
		Total exosome isolation reagent (Invitrogen, United States)	Serum; 208 AD patients; 228 HCs; 20 VAD patients; 30 PDD patients	miR-135a	0.981	Upregulated	Yang et al. (2018)	Also distinguish AD from HCs/PDDs/VaDs patients
				miR-193b	0.798	Downregulated		
				miR-384	0.87	Upregulated		
	PD	ExoQuick exosome precipitation solution (System Biosciences,CA, United States)	Plasma; 39 PD patients; 40 HCs	DJ-1	0.703	Upregulated	Zhao et al. (2018a)	
				α-synuclein	0.654	Upregulated		
	Immune-Mediated Demyelinating Disease	Ultracentrifugation	CSF; 5 immune-mediated demyelinating disease patients and 5 underwent lumbar puncture because of abnormal intracranial pressure patients	hsa_circ_0087862	1	Upregulated	He et al. (2019)	
				hsa_circ_0012077	1	Upregulated		
Digestive system	AH	Ultracentrifugation	plasma; 25 AH patients and 18 HCs	sphingosine	0.85	Upregulated	Sehrawat et al. (2021)	
				sphinganine	0.85	Upregulated		
				Sphingosine 1-phosphate	0.81	Upregulated		
				C14:0 ceramide	0.82	Upregulated		
				C16:0 ceramide	0.83	Upregulated		
				C18:1 ceramide	0.76	Upregulated		
				C18:0 ceramide	0.741	Upregulated		
				C20:0 ceramide	0.73	Upregulated		
				C22:0 ceramide	0.74	Upregulated		
				C24:1 ceramide	0.8	Upregulated		
				C24:0ceramide	0.72	Upregulated		
Urinary system	RF	PEG-based method (PEG 6000, Sigma)	Urine; 35 RF patients; 20 HCs	miR-29c	0.8333	Downregulated	Lv et al. (2018)	
				miR-21	0.7639	Upregulated		
	FSGS	ExoQuick exosome precipitation (System Biosciences, Mountain View, CA, United States)	Urine; 8 FSGS children patients and 5 MCD children patients	mir-193a	0.85	Upregulated	Huang et al. (2017)	
Circulatory system	ACS	Ultracentrifugation	Serum; 34 AM patientsI, 31 UA patients, and 22HCs	mir-122-5p	0.924/0.765	Upregulated	Ling et al. (2020a)	Distinguish AMI/UA patients from HCs(AUC:0.924/0.765)
		Ultracentrifugation		miR-21	0.824/0.8422	Downregulated	Ling et al. (2020b)	Distinguish AMI/UA patients from HCs(AUC:0.8422/0.824)
				miR-126	0.8489/0.7815	Upregulated		Distinguish AMI/UA patients from HCs(AUC:0.8489/0.7815)
	HFrEF	Exosome isolation reagent (R11064.5, RiboBio, Guangzhou, China)	Serum; 28 HFrRF patients; 30HCs	miR-92b-5p	0.844	Upregulated	Wu et al. (2018)	
Cancer	PDAC	AC Electrokinetic isolation	Serum; 192 PDAC patients; 100 healthy donors	Glypican1	1	Upregulated	Lewis et al. (2018)	
	BC	Combination of centrifugation and ultracentrifugation/Exoquick-TC reagent (System Biosciences, Mountain View, CA, United States)	Plasma; 16 HCs and 16 BC patients	miR-1246	0.69	Upregulated	Hannafon et al. (2016)	
				miR-21	0.69	Upregulated		
	PCA	Ultracentrifugation	Urine; 19 HCs and 28 PCA patients	miR-196a-5p	0.73	Downregulated	Rodriguez et al. (2017)	
				miR-501-3p	0.69	Downregulated		
		Ultracentrifugation	Serum; 20 HCs and 21 BPH patients, and 50 PCa patients	EphrinA2	0.9062	Upregulated	Li et al. (2018a)	Distinguish PCA from BPH patients
		ExoRNeasy Serum/Plasma Maxi Kit (Qiagen 77064)	Serum; 141 PCa patients, 170 BPH patients and 30 HCs	Combination of CDC42, IL32, MAX, NCF2, PDGFA and SRSF2 mrna	0.948	Upregulated	Ji et al. (2021)	Distinguish BPH patients from HCs(AUC:0.851)
	HCC	Exosome isolation kit (Invitrogen, United States)	Plasma; 5 HCs and 5 HCC patients	Combination of tRNA-ValTAC-3,tRNA-GlyTCC-5,tRNA-ValAAC-5 and tRNA-GluCTC-5	-	Upregulated	Zhu et al. (2019)	
		ExoQuick Exosome Precipitation Solution (System Biosciences, Mountain View, CA)	Serum; 28 non‐HCC patients; 28 HCC patients without posttransplant; 43 HCC patients with early 22 HCC recurrence and HCC patients with late HCC recurrence	miR-92b	0.925	Upregulated	Nakano et al. (2019)	Early recurrence diagnosis after LDLT
		ExoQuick Exosome Precipitation Solution (System Biosciences)	Plasma; 180 HCs; 180 HCC patients; 180 CH patients	ENSG00000248932.1	0.794	Upregulated	Lu et al. (2020)	Combination of ENSG00000248932.1 、ENST00000440688.1 and ENST00000457302.2 (AUC:0.838)
				ENST00000440688.1	0.571	Upregulated		
				ENST00000457302.2	0.538	Upregulated		
	CC	Ultracentrifugation	cervicovaginal lavage; 30 HCs(HPV-); 30 CC patients; 30 CC patients (HPV+)	lncRNA HOTAIR and MALAT1	-	Both upregulated	Zhang et al. (2016)	
				lncRNA MEG3	-	Downregulated		
		Ultracentrifugation	Plasma; 58 CC patients and 76 HC patients	let-7b-3p	0.792	Downregulated	Min et al. (2019)	combination of let-7b-3p、miR-145-3p、miR-139-3p (AUC:0.927)
				miR-150-3p	0.686	Downregulated		
				miR-145-3p	0.679	Upregulated		
				miR-139-3p	0.692	Upregulated		
	GC	-	Serum; 126 GC patients; 120 healthy donors	Lnc RNA HOTTIP	0.827	Upregulated	Zhao et al. (2018b)	
		Ultracentrifugation	Serum; 108 GC patients; 108 healthy donors	miR-15p-3p	0.82	Upregulated	Wei et al. (2020a)	
	CRC	Total Exosome Isolation Kits (4478359 and 4478360) (Invitrogen, NYC, United States)	Serum; 135 CRC patients, 35 BID patients and 45 HCs	hsa-circ-0004771	0.86/0.88	Upregulated	Pan et al. (2019)	Distinguish I/II stages/all stage CRC patients from HCs(AUC:0.86/0.88); Distinguish I/II CRC patients from BID patients (AUC:0.81)
		Ultracentrifugation	faecal; 48 CRC patients; 16 HCs	CD147	0.903	Upregulated	Zhang et al. (2023)	Combination of CD147 and A33 (AUC:0.913)
				A33	0.904	Upregulated		
		Ultracentrifugation	Plasma; 163 CRC patients 51 adenoma (non-HCC) patients and 46 HCs	Epcam-CD63	0.9	Upregulated	Wei et al. (2020b)	
	Ovarian Cancer	Microfluidics	Plasma; 15 OVCA patients; 5 HCs	CA125	1	Upregulated	Zhao et al. (2016)	
				EpCAM	1	Upregulated		
				CD24	0.91	Upregulated		
		Ultracentrifugation	Plasma; 20 OVCA patients; 10 non-cancer controls	FRα	0.995	Upregulated	Zhang et al. (2019a)	
				EpCAM	1	Upregulated		
				CD24	1	Upregulated		
		PEG/DEX aqueous two-phase system	Plasma; 21 HCs and 34 papillary serous carcinoma ovarian cancer patients	miR-4732-5p	0.889	Upregulated	Liu et al. (2021)	
	NSCLC	Exosome precipitation solution (ExoQuick™, SBI, USASBI, United States)	Serum; 72 NSCLC patients and 47 HCs	miR-17-5p	0.738	Upregulated	Zhang et al. (2019b)	
		Immunoaffinity magnetic beads	Plasma; 13HCs and 47 NSCLC patients (34 AC and 13 SCC patients)	Combination of miR-181-5p and miR-361-5p/Combination of miR-320b and miR-10b-5p、Combination of let-7b-5p,let-7e-5p,miR-24-5p and miR-21-5p	0.936/0.911、0.899	miR-181-5p:upregulated	Jin et al. (2017)	Distinguish AC/SCC patients from NSCLC patients (AUC:0.936/0.911); Distinguish NSCLC patients from HCs(AUC:0.899)
						miR-361-5p:upregulated		
						miR-320b:upregulated		
						miR-10b-5p:downregulated		
						let-7b-5p:upregulated		
						let-7e-5p:downregulated		
						miR-24-3p:upregulated		
						miR-21-5p:downregulated		

Abbreviation: HCs, Healthy Controls; TB, Tuberculosis; PTB, Pulmonary Tuberculosis; TBM, Tuberculous Meningitis; AMCI, Amnestic Mild Cognitive Impairment; AD, Alzheimer’s disease; VAD, Vascular Dementia; PDD, Parkinson’s disease; PD, Parkinson’s Disease; AH, Alcoholic Hepatitis; AMI, Acute Myocardial Infarction; UA, Unstable Angina; RF, Renal Fibrosis; FSGS, Focal Segmental Glomerulosclerosis; MCD, Minimal Change Disease; ACS, Acute Coronary Syndrome; HFrEF, heart failure with reduced ejection fraction; PDAC, Pancreatic Ductal Adenocarcinoma; BC, Breast Cancer; PCA, Prostate Cancer; BPH, Benign Prostatic Hyperplasia; CC, Cervical Cancer; CRC, Colorectal Cancer; BID, benign intestinal diseases; CC, Colon cancer; GC, gastric cancer; HCC, hepatocellular carcinoma; CH, Chronic Hepatitis; NSCLC, non-small cell lung cancer; AC, adenocarcinoma; SCC, squamous cell carcinoma.

Taking into account these aforementioned advantages, exosomes have demonstrated remarkable diagnostic capabilities surpassing those of conventional biomarkers. Nevertheless, the time and cost associated with their extraction remains a topic of debate when compared to automated detection instruments. In order to fully harness the potential of exosomes in disease diagnosis, it is imperative to enhance their sensitivity and diminish the costs associated with their detection. With ongoing advanced research, it is anticipated that more cost-effective and commercially accessible exosome detection kits will become available in the foreseeable future.

#### Potential vaccine for disease prevention

The use of exosomes as vaccines holds great promise in the field of immunology. Exosomes are small extracellular vesicles that are secreted by living organisms and can play an important role in intercellular communication. As a vaccine, exosomes offer several advantages over traditional vaccines: Low toxicity and immunogenicity compared to many live vaccines and influenza vaccines; High efficiency to enter cells and release antigens for the activation and improve vaccine efficacy; Exosomes can be manipulated in terms of their composition and characteristics, allowing for flexible adjustment to transport and deliver a variety of vaccines (Santos and Almeida, 2021).

The role of communication between immune cells (macrophages, dendritic cells (DC), T cells and B cells, etc.) in the immune system is well established. Exosomes act as trucks for intercellular communication, and thus it has been increased more attention in the field of vaccine research. Exosomes isolated from LPS stimulated macrophages can stimulate the secretion of IFN-γ that appear stronger *in vivo* in mice in response to hepatitis B virus surface antigen (Jesus et al., 2018). DC derived exosome vaccine has great potential in metastatic melanoma and hepatocellular carcinoma (Escudier et al., 2005; Lu et al., 2017). And exosomes derived from M1 macrophages enhanced the activity of lipid calcium phosphate (LCP) nanoparticle encapsulated Trp2 vaccine and induced stronger antigen-specific cytotoxic T-cell responses through enzyme-linked immunospot (ELISPOT) assay (Cheng et al., 2017). Exosomes secreted by CD4 + T cells can significantly contribute to enhanced B cell activation and increased antibody production in the face of hepatitis B surface antigen in a dose-dependent manner (Lu et al., 2019).

In general, tumor cells can release exosomes to influence the immune system, suggesting that tumor derived exosomes may also serve as vaccines for cancer immunotherapy. Tumor derived exosomes enhance DC immunogenicity via exosome anchored peptide coated functional domains of a potent adjuvant (HMGN1) (Agrawal et al., 2017). Tumor peptide pulsed DC derived exosomes have also been identified to cause specific cytotoxic T lymphocytes as well as inhibit established mastocytoma and breast cancer growth (Whitehead et al., 2009). Tumor derived HSP70/bag-4 surface positive exosomes can stimulate NK cells to release granzyme B, which in turn triggers tumor cell apoptosis (Esteves et al., 2022). They loaded immunogenic cell death agents into α-Whey protein engineered MDA-MB-231/Luc cell-derived exosomes *in vitro* then activate DCS, ultimately thereby contributing to type one conventional DC maturation and CD8 + T cell activation *in vitro* (Huang et al., 2022).

Unlike the usual thinking of screening neoantigens from tumor cells, they derived ESAT6 from *Mycobacterium tuberculosis* and found that ESAT6 + exosomes could significantly contribute to the inhibition of neoantigens in B16 tumor bearing mice (Koyama et al., 2016). The number of T cells could be dose dependently stimulated to increase when the conjugation of Ag85B and ESAT6 to ubiquitin in exosomes was followed by *in vivo* experiments (Cheng and Schorey, 2016). Admittedly, Toxoplasma gondii exosomes could modulate macrophages to secrete inflammatory factors and elicit humoral and cellular immune responses in mice, suggesting that exosomes could serve as potential vaccine candidates against toxoplasmosis, bacteria, fungi, and viruses as well as cells after their infection can secrete exosomes (Li et al., 2018b), it is a vexing matter how to isolate and purify exosomes secreted by cells and pathogens when conducting research.

Overall, the use of exosomes as vaccines or vaccine delivery systems has significant potential to revolutionize the field of immunology and disease treatment, offering a safe and effective alternative to traditional vaccines. With ongoing research and development, we can expect to see more widespread use of exosome-based vaccines in the near future.

#### Engineered exsosomes as delivery system

Exosomes have emerged as a promising drug delivery vehicle due to their unique properties. Firstly, they are naturally secreted by cells and are involved in intercellular communication, making them well-tolerated by the body with minimal toxicity (Yim et al., 2016). Secondly, they can easily penetrate cell membranes due to their small size, allowing them to efficiently deliver drugs to target cells (Hikita and Oneyama, 2022). Thirdly, they can be loaded with a wide range of therapeutic agents, such as proteins, nucleic acids, and small molecules, by modifying their surface or cargo content (Jiang et al., 2017). Additionally, they can protect their cargo from degradation and immune clearance, thus improving the stability and bioavailability of the drugs (Alhasan et al., 2014). Finally, they have the ability to target specific cells or tissues through their surface proteins, allowing for more targeted drug delivery (Salunkhe et al., 2020).

Many of the current methods for collecting exosomes are based on isolating exosomes from media obtained by classical cell culture methods. However, these methods have several limitations. For instance, they typically require large input volumes, obtained from large-scale cultures of cells, which can be time-consuming. Additionally, these methods often yield low concentrations of exosomes. In recent studies, a three-dimensional (3D) culture system using a hollow fiber bioreactor has shown potential advantages over conventional culture systems for exosome production. This 3D culture system offers higher capacity and lower cost compared to traditional methods, such as 2D-exo. It is important to note that the cargo contents of exosomes produced in 2D and 3D environments differ from each other (Haraszti et al., 2018; Wang et al., 2021). However, it should be acknowledged that 3D-exo still has some inherent limitations.

Exosomes play a crucial role as carriers for targeted drug delivery and therapy, and their purity directly impacts the function of recipient cells. Hence, in the realm of clinical diagnosis and treatment, researchers continually raise their expectations for the isolation and purification techniques of exosomes. It is important to consider the effect of rotor type, centrifugation time, and speed on exosomes since most separation methods involve centrifugation (Cvjetkovic et al., 2014). Table 2 provides a summary and comparison of several commonly used exosome isolation methods, highlighting their principles, advantages, and disadvantages. To optimize exosome production, scientists have explored the cultivation of umbilical cord-derived mesenchymal stem cells in a scalable, microcarrier-based, three-dimensional (3D) culture system. The combination of tangential flow filtration (TFF) with 3D mesenchymal stem cell culture enhances exosome production (referred to as 3D-TFF-exo) by 140-fold and improves siRNA delivery to neurons by 7-fold compared to 2D-exo (Haraszti et al., 2018). Furthermore, recent studies have demonstrated that the synergistic use of multiple methods is also beneficial for exosome separation and purification (Bellotti et al., 2021; Visan et al., 2022).

**TABLE 2:** The limitations and advantages of exosome isolation method (Yang et al., 2020a).

Isolation method	Principle	Advantages	Limitations
Ultracentrifugation	According to the difference in sedimentation velocity of different substances	Gold standard; High purity	Long time required; Expensive equipment; Low yield; Risk of exosome rupture
Immune affinity capture	According to the antigens on the surface of exosomes	Strong specificity; Isolation of specific exosome subtypes; High-cost	Low yield; High purity; High cost; Cancer heterogeneity influences antibody recognition
Precipitation	According to hydrophobic protein and lipid fractions of exosomes	High yield; Operation is simple; Short time	Low purity
Microfluidic	Immunoaffinity, sieving, and trapping exosomes on porous structures	High sensitivity; High throughput analysis	Inadequate quality control
Size-based filtration	According to the molecular weight cut-off of ultrafiltration membrane	Short time; Operation is simple	Risk of exosome rupture
Siza-based chromatography	The pore size of the gel pores was plotted against the molecular size of the sample	Maintaining the integrity and bioactivity of exosomes; Operation is simple	Risk of exosome rupture; Device unique

Exosomes are commonly used for delivering RNA or drugs, with two methods available for loading them, namely, preload and afterload. Preload involves delivering cargo to cells through transfection or co-incubation, followed by exosome collection. Meanwhile, afterload requires collecting exosomes from parental cells and then inducing RNA and/or drug entry by co-incubation, electroporation, or ultrasound (Ha et al., 2016). Electroporation, is found to result in extensive siRNA aggregates with much lower efficiency, highlighting the need for alternative methods in preparing siRNA loaded extracellular vesicles (Kooijmans et al., 2013). Despite the increase in exosome size after cargo loading by most methods, the protein marker remains stably expressed, demonstrating its applicability (Haney et al., 2015; Kim et al., 2016; Wang et al., 2019). Then, Improving the efficiency of exosome loading is still a priority, with previous studies showing that sonication has better loading efficacy than co-incubation and electrotransformation, making it a frontrunner in the field of loading drugs to exosomes (Haney et al., 2015; Kim et al., 2016; Gao et al., 2021; Tenchov et al., 2022). Kit-based methods have been successfully used to transfect exogenous nucleic acids into exosomes (Nie et al., 2020). Treatments such as saponin-assisted, freeze-thaw cycle, and extrusion may change the exosome morphology, biomarkers, or biological characteristics that may limit exosome development (Fuhrmann et al., 2015). Table 3 summaries the loading method of exosome.

**TABLE 3:** The loading method of exosome.

	Loading method	Donor cell	Cargo	Exosome isolation method	Disease	Reference
Pre-secretory loading	transfection	HEK 293T cell	let-7a	Ultracentrifugation	breast cancer	Ohno et al. (2013)
		γδ T cell	miR-138	ExoQuick EV precipitation solution (SBI System Biosciences, CA, United States of America)、ultracentrifugation	Oral Squamous Cell Carcinoma	Li et al. (2019)
		HEK 293T cell	siBCR-ABL	centrifugation	Chronic Myelogenous Leukemia	Bellavia et al. (2017)
		HEK 293T cell	anti-miR214	centrifugation	Chemoresistance to Cisplatin in Gastric Cancer	Wang et al. (2018)
	Co-incubation	HEK 293T cell	Imatinib	centrifugation	Chronic Myelogenous Leukemia	Bellavia et al. (2017)
Post-secretory loading	Electroporation	HEK 293T cell	siTPD52	Ultracentrifugation	Breast Cancer	Limoni et al. (2019)
		RBCs	CRISPR–Cas9 genome editing system/miR-125b-ASO	Ultracentrifugation	leukemia/breast cancer	Usman et al. (2018)
		Macrophages	Superparamagnetic iron oxide nanoparticle and curcumin	size-based filtration	glioma	Jia et al. (2018)
		Macrophages	Gemcitabine and Deferasirox	Ultracentrifugation	Chemoresistant Pancreatic Cancer	Zhao et al. (2021)
		human lung spheroid cells	MRNA encoding the coronavirus 2 (sars-cov-2) spike (s) protein	size-based filtration	severe acute respiratory coronavirus 2	Popowski et al. (2022)
	Co-incubation	tumor cells	curcumin	Ultracentrifugation	Brain Inflammatory Diseases	Zhuang et al. (2011)
		mature bovine milk	anthocyanidins and paclitaxel	Ultracentrifugation	ovarian cancer	Aqil et al. (2017)
		tumor cells	hsiRNAs	Ultracentrifugation + Size exclusion chromatography	Huntingtin disease	Didiot et al. (2016)
		HEK 293T cell	Romidepsin	size-based filtration	cancer	Si et al. (2020)
		tumor cells	curcumin	Ultracentrifugation	pulmonary inflammation	Sun et al. (2010)
	Sonication	Macrophages	paclitaxel	ExoQuick-TC™ Kit (System BioSciences; Mountain View, CA)	lung carcinoma	Kim et al. (2016)
		Macrophages	catalase	Ultracentrifugation	Parkinson’s Disease Therapy	Haney et al. (2015)
		Macrophages	paclitaxel	Ultracentrifugation	cancer	Wang et al. (2019)
		Macrophages	Berberine	Ultracentrifugation	Spinal cord injury	Gao et al. (2021)
	saponin	HMSCs、HUVECs、MDAs	porphyrins	Ultracentrifugation	—	Fuhrmann et al. (2015)

To better target recipient cells *in vitro* and disease areas *in vivo*, researchers have developed exosome targeting technology, which can further enhancing the targeting effects of the designed exosomes and thus allow more functional application of exosomes in biomedicine.

#### Pre-modification: genetic engineering

Pre-modification involves utilizing the cellular expression machinery and natural exosome generation mechanism, allowing targeting sites to be conjugated to lamp2b. HEK293T cells were modified to produce a fusion protein consisting of Lamp2b, an exosomal protein, and a fragment of Interleukin 3 (IL3). The elevated expression of the IL3 receptor (IL3-R) in CML blasts, as compared to normal hematopoietic cells, allows it to serve as a specific receptor target in a cancer drug delivery system (Bellavia et al., 2017). In HEK293T cells, siRNA was loaded into extracellular vesicles (EVs) for delivery to SKBR3 cells. The genetically stable HEK293T cells efficiently expressed DARPin G3 on EV surface, enabling specific binding with HER2/Neu. The designed EVs successfully delivered TPD52-targeting siRNA to SKBR3 cells, leading to a remarkable 70% downregulation of gene expression (Limoni et al., 2019). The researchers utilized chondrocyte-targeting exosomes to deliver miR-140 for osteoarthritis (OA) treatment. They achieved this by fusing a chondrocyte-affinity peptide (CAP) with lysosome-associated membrane glycoprotein 2b on exosomes. CAP-exosomes efficiently encapsulated miR-140 and selectively entered chondrocytes, delivering the cargo *in vitro* (Liang Y. et al., 2020). In a similar manner, targeting can be accomplished by genetically modifying dendritic cells to express Lamp2b (Alvarez-Erviti et al., 2011). However, the presence of endosomal proteases during exosome formation poses a challenge as it leads to the degradation of peptides, making it difficult to achieve the desired yield of peptide-functionalized exosomes. Additionally, some studies have fused the GE11 tumor targeting polypeptide, a ligand for EGFR, to the PDGFR terminus (Ohno et al., 2013).

#### Post-modification: covalent modification and non-covalent modification

Post-modification is critical due to the complex process of exosome production and the variability in protein expression across different levels. Covalent coupling strategies involving chemical cross linkers and noncovalent coupling strategies utilizing hydrophobic or charge interactions are common methods for coupling targeting ligands onto exosome membranes (Liang et al., 2021). Conventional chemical reactions involve fluctuations in temperature or pressure and inappropriate changes in osmotic pressure, leading to membrane disruption or aggregation of extracellular vesicles. Click chemistry reactions are efficient as they can be performed in organic solvents and aqueous buffer solutions, with shorter reaction times. Coupling reactions do not affect the size of extracellular vesicles or their uptake into target cells, indicating that surface modifications of extracellular vesicles have appropriate reaction conditions. The exosomes, crosslinked with alkynyl groups using carbodiimide chemistry, were conjugated with the azide fluoro545 model. This coupling process does not alter the size of exosomes, nor does it affect the degree of adhesion/internalization between exosomes and receptor cells. These findings suggest that the reaction conditions employed for the modification of exosomes are gentle, preserving both the structural integrity and functional properties of the exosomes (Smyth et al., 2014). Researchers successfully achieved the loading of superparamagnetic iron oxide nanoparticles (SPIONs) and curcumin (Cur) into exosomes. They further performed click chemistry to conjugate the exosome membrane with neuropilin-1 targeting peptides (RGERPPR, RGE). This innovative approach resulted in the development of targeted exosomes specifically designed for glioma, offering combined imaging and therapeutic capabilities (Jia et al., 2018). The non-covalent modification of exosomes involves the attachment of modifiers to the surface structure of exosomes through non-covalent interactions. These modifiers can encompass lipid structures, glycosides, proteins, nucleic acids, and more. One article discusses the surface modification of exosomes using cationized pullulan to improve their liver targeting capabilities and enhance exosome-based drug delivery (Tamura et al., 2017). In another study, researchers explore a combined treatment approach employing a pH-sensitive fusogenic peptide and cationic lipids to enhance the cytosolic delivery of exosomes (Nakase and Futaki, 2015). Additionally, the use of magnetic exosomes for targeted drug delivery to specific tumor areas is investigated, potentially enhancing the effectiveness of cancer treatment (Qi et al., 2016). This study underscores the significant potential of this strategy in the field of tumor treatment.

**FIGURE 1:**
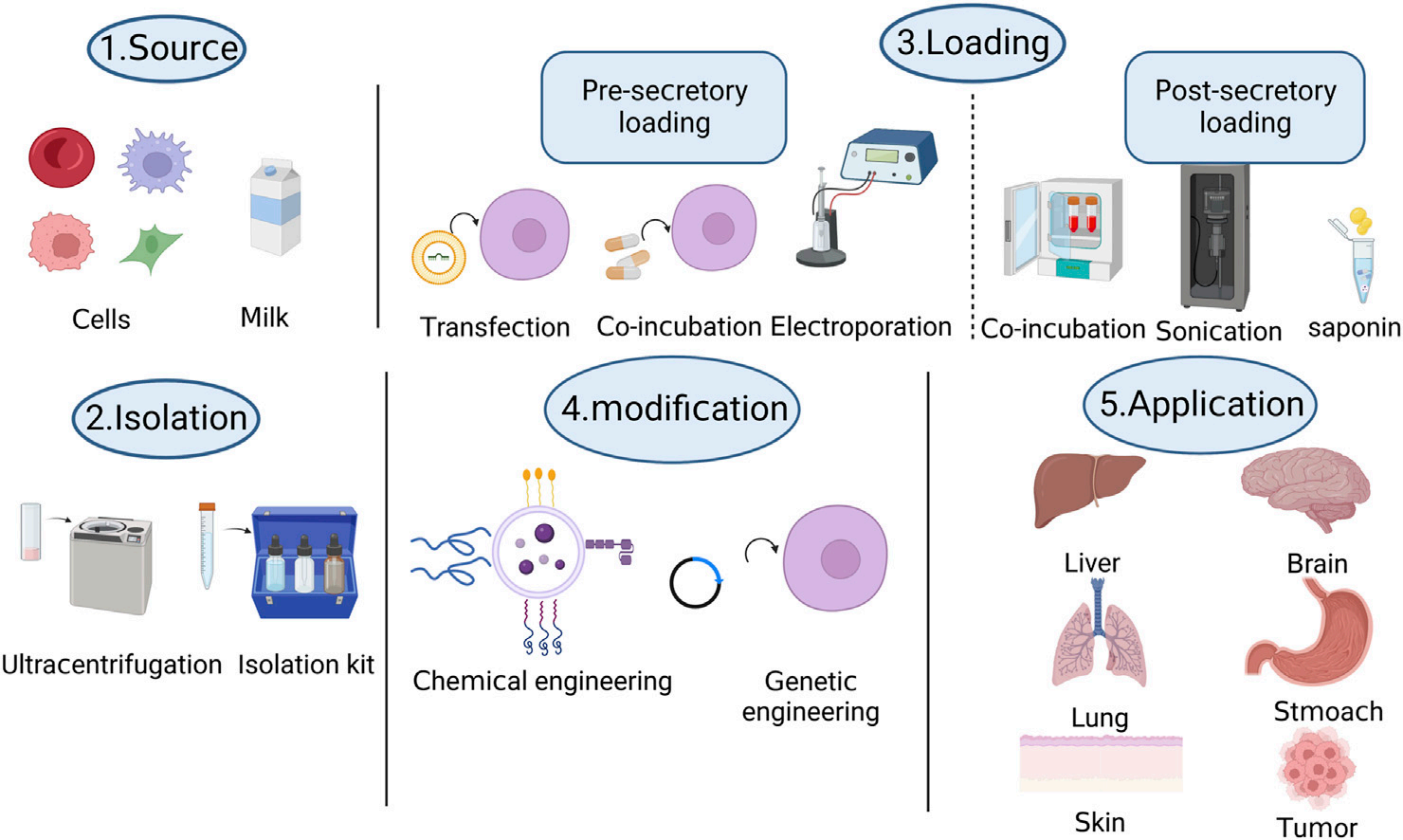
Visualizing the production process of engineered exosomes through a schematic diagram.

Fundamentally, challenges such as storage, extraction, quantification, modification, and loading of exosomes hinder the development of exosomes as drug or nucleic acid delivery systems. Figure 1 shows the production process of engineered exosomes. Additionally, the *in vivo* transfer of exosomes and the delayed mechanism of cargo delivery through body fluids and to specific targets necessitate further consideration. The clinical applications of drug delivery vehicles, which come with their own set of challenges and limitations, require more exploration and standardization. We hope that by fostering collaboration among researchers from different disciplines, such as clinicians, cell biologists, and computer experts, we can advance exosomes hand-in-hand into the realm of clinical applications.

### Exosomes as carriers of drug for enhanced drug efficiency

Small molecule drugs spearheaded the therapeutic landscape for many years, but their shortcomings also slowly unfold. The first most hindering route of drug administration is resistance mechanisms, including gene mutation, amplification, CSCs, efflux transporters, apoptosis dysregulation, and autophagy, et, which are encountered by almost all targeted anticancer drugs after some time in clinical use (Zhong et al., 2021a). Oral intake is the most commonly used route of administration because of ease of administration, high patient compliance, low cost, and flexibility in dosage form design, with compounds of low solubility resulting in poor absorption and bioavailability, insufficient solubility for intravenous administration, and development challenges resulting in increased development cost and time and a burden that is transferred to patients (frequent bolus administration) (Savjani et al., 2012). Nanoparticle—and microparticle based systems have been used to overcome the challenge of solubility, enabling small molecules to be trafficked to their site of action (Amreddy et al., 2018). Meanwhile, exosomes, have been largely developed for drug loading, have attracted increasing attention as a potentially effective drug delivery system in disease treatment due to their good biocompatibility, high stability, and good targeting.

Cancer has always been a killer of people’s continuous confrontation, and its high case fatality rate and low survival rate remain unsatisfactory. One of the reasons is that most of the current antineoplastic drugs have the disadvantages of strong cytotoxicity, poor biocompatibility and low bioavailability, leading to poor efficacy of chemotherapy.

Paclitaxel (PTX), as the first natural anticancer drug approved by FDA, has limited its clinical application due to its poor resources and low water solubility problems. Different types of nano carriers, including lipids, proteins, polymers, solid nano lotion and hybrid systems, have been reported to be used to wrap paclitaxel for cancer treatment (Khalifa et al., 2019). Macrophage derived exosomes exerted tumor growth inhibitory properties by encapsulating paclitaxel, whether *in vivo* or *in vitro*, and it was found that the encapsulated exosomes did not induce cell degeneration or necrosis in major organs (Kim et al., 2016; Wang et al., 2019). They made a bold guess that the incorporation of PTX into exosomes could not only increase their solubility but also overcome PGP mediated drug efflux, although the specific mechanism is still not well understood.

Curcumin, as a traditional Chinese herbal medicine, has anti-tumor, anti oxidant and anti-inflammatory effects, and has some efficacy in the treatment of mild and toxic COVID-19 infectious patients (Saber-Moghaddam et al., 2021). Relevant literature shows that Curcumin loaded POCA4C6 micelles have proved the rationality of further efforts to develop CPM as a treatment for breast cancer, including more detailed research on how POCA4C6 and Curcumin synergistically inhibit tumor growth and destroy BCSC (Chen et al., 2017a). Prof. Zhang’s team encapsulated curcumin into tumor or macrophage derived exosomes, which could be efficiently delivered to the brain or lungs and acted as anti-inflammatory (Sun et al., 2010; Zhuang et al., 2011). In addition, a highly efficient surface labeling technique to generate mAb exosomes loaded with romidepsin was developed, and finally its unique targeting to cancer cells and antitumor activity was confirmed (Si et al., 2020).

Doxorubicin, an antitumor broad-spectrum antibiotic used to inhibit the synthesis of RNA and DNA, has the strongest inhibitory effect on RNA. Previous literature has reported that chemically responsive nanoparticles have the potential to induce apoptosis in MCF-7 cells by enhancing DOX uptake (Zhang et al., 2022a). Yanget al used a zebrafish (*Danio rerio*) model, which have a fully developed and functioning BBB that has similar properties to the human BBB, to examine the efficacy of brain endothelium derived exosomes to deliver the antitumor drug doxorubicin through the blood-brain barrier (Yang et al., 2015). Doxorubicin loaded into exosomes derived from other cells similarly exhibits antitumor properties (Tian et al., 2014; Wei et al., 2019), and to promote tumor cell targeting, it can be facilitated by engineering the imDCs to express a well characterized exosomal membrane protein (lamp2b) fused to αv integrin-specific iRGD peptide (CRGDKGPDC) (Tian et al., 2014).

Loading of celastrol with exosomes improves its antitumor properties, possibly by inhibiting NF- κB The pathway (Aqil et al., 2016). Researchers have loaded aspirin into exosomes from tumor cells and gemcitabine and deferasiro co loaded into exosomes from M1 macrophages, thus showing great potential in the field of exosome loading drugs against cancer (Tran et al., 2019; Zhao et al., 2021).

The blood-brain barrier can leave brain tissue less damaged or even not by harmful substances in the circulating blood, thereby maintaining the basic stability of the brain tissue internal environment. Exosome loading of drugs has potential not only in the anti-tumor field, but also in the treatment of brain diseases due to the fact that it can cross the blood-brain barrier to reach the lesion site. In fact, most drugs cannot pass through the blood-brain barrier (Pandit et al., 2020). Exosomes are readily taken up by neuronal cells *in vitro*,A comparable number of exosomes were detected in the brains of PD mice after intranasal administration (Haney et al., 2015). Loading this bullet point of curcumin into exosomes, and then conjugating the exosome membrane with neuropilin-1-targeted peptide (RGERPPR,RGE)neuropilin-1-targeted peptide or the C (rgdyk) peptide had been conjugated to the exosome surface by click chemistry (Jia et al., 2018), will realize the breakthrough blood-brain barrier for the treatment of brain diseases. Loading berberine with exosomes induced overactivation of microglia, the transition from pro-inflammatory M1 type microglia to anti-inflammatory M2 type microglia, reduced neuronal apoptosis after SCI, and promoted motor function recovery (Gao et al., 2021).

The last barrier against drug treatment of disease is the gastrointestinal barrier. Either human or bovine milk exosomes can pass through the gastrointestinal barrier, making them promising oral drug delivery tools. On the one hand, the function of human milk exosomes has been largely demonstrated, but research on therapeutics to load them with drugs is still in the rising stage, and on the other hand, curcumin, paclitaxel, anthocyanins and doxorubicin can be loaded into bovine milk exosomes using appropriate solvents (Agrawal et al., 2017; Zhong et al., 2021b). This notion is not unexpected with the acidic pH could reduce degradation of exosome associated proteins (Cheng et al., 2019).

Overall, relative to free drugs, on the one hand, after reaching the disease site, drugs after loading into exosomes show stronger therapeutic effects, drug loading and longer duration (Sun et al., 2010; Kim et al., 2016) on the other hand, exosomes can cross the blood-brain barrier and gastrointestinal barrier as well as avoid being trapped by the reticuloendothelial system it is clear that exosomes must walk farther along the path of drug loading, although its starting point is later than liposomes.

### Exosomes as loading system for RNA delivery

RNA therapeutics offer several advantages, such as rapid production, long-term effects, and a lack of genotoxicity risk, which make them particularly useful for treating rare diseases (Whitehead et al., 2009). In contrast, exosomes have several unique characteristics, such as their small size, low immunogenicity, and customizability, which make them attractive for RNA delivery. While further research is still needed to fully explore the potential of exosomes for clinical applications, current investigations show promise for their use in the future.

miRNA is small non-coding RNA molecule that plays a critical regulatory role in gene expression (Bartel, 2009). One advantage of using miRNA-based therapies is their high specificity, allowing for targeted treatment with minimal off-target effects, however, there are also some limitations of using miRNAs as therapeutics, including delivery challenges and potential immune responses (Zhang et al., 2013). Recent research has focused on using extracellular vesicles, specifically exosomes, as a delivery system for miRNA-based therapies. There have been several studies that have demonstrated the potential of using exosomes as a delivery system for miRNA-based therapies. For instance, However, some polymeric nanoparticles that contain miR-124 are unable to migrate to the substantia nigra in order to enhance the function of dopamine neurons, so researchers have found that loading miR-124 into exosomes derived from human cord blood monocytes allows for effective transportation of miR-124 to the substantia nigra and protection of dopamine neurons (Esteves et al., 2022). Other studies have utilized exosomes collected from γδT cells, which were loaded with miR-138 and had a suppressive effect on oral squamous cell carcinoma (Li et al., 2019). Targeted modification of exosomes can also be achieved through the expression of the platelet-derived growth factor receptor’s transmembrane domain fused with a GE11 peptide in HEK293T cells (Ohno et al., 2013). Moreover, administering exosomes containing anti-mir-214 can reverse the resistance of gastric cancer to cisplatin and induce apoptosis, offering a promising approach for the treatment of cisplatin refractory gastric cancer in the future (Wang et al., 2018).

Antisense oligonucleotides (ASOs) are rapidly gaining recognition as a potent tool in gene therapy that allows for targeted regulation of gene expression at the RNA level (Kim and Rossi, 2007). However, the challenge of successfully delivering ASOs as nucleic acid therapeutics has been addressed by the discovery of exosomes. Researchers have utilized exosomes secreted by erythroid cells for the delivery of anti-mir-125b ASOs, leading to effective treatment of tumors with minimal interference from other genes (Usman et al., 2018). Moreover, exosomes loaded with anti-mir-210-asos, delivered by IFN- γ exosomes derived from stimulated human umbilical cord tissue-derived MSCs, were able to effectively alleviate symptoms of psoriasis (Zhang et al., 2022b). Exosomes capable of delivering ASOs targeting STAT6 have shown remarkable success in treating colorectal and hepatocellular carcinoma in syngeneic models, leading to inhibition of tumor growth and complete remission in 50%–80% of cases. Furthermore, these exosomes show the potential to reprogram immunosuppressive tumor-associated macrophages from a pro-tumor M2 phenotype to a pro-inflammatory M1 phenotype (Kamerkar et al., 2022).

The use of siRNA for targeted gene silencing has gained much attention due to its high specificity, strong efficiency, and minimal adverse effects (Dua et al., 2019). However, siRNA-based therapies also have some limitations, including potential off-target effects and poor delivery into cells. Off-target effects can lead to unintended gene knockdown, resulting in cytotoxicity or harmful side effects. Delivery into cells is another significant hurdle, as siRNAs are unable to cross the cell membrane efficiently (Kanasty et al., 2013; Subhan and Torchilin, 2019). To address these challenges, exosome-based delivery systems have emerged as a promising approach to the safe and efficient delivery of siRNA.Researchers have developed exosomes that express specific targeting molecules on their surface. For example, HEK293T cells can generate exosomes containing the targeting molecules DARPinG3 and lamp2b, fused with the fragment of IL3 produced on the surface of exosomes. When loaded with sitpd52, these exosomes are capable of downregulating target gene expression by up to 70% or inhibiting cancer cell growth *in vitro* and *in vivo* when loaded with imatinib or siBCR-ABL (Bellavia et al., 2017; Limoni et al., 2019). Unmodified exosomes may also have translational potential, studies have shown that exosomes loaded with hsiRNAs have better drug distribution relative to free hsiRNAs (Didiot et al., 2016).

To enhance the biostability of conventional siRNA, different chemical modifications have been utilized with considerable success. Alternatively, the absence of termini in circRNA offers a promising solution for developing exonuclease-resistant siRNA through a different approach. A study has shown that EVs engineered with rabies virus glycoprotein (RVG) have been effective in delivering therapeutic circRNAs specifically to the brain, resulting in functional recovery in non-human primate models of ischemic stroke (Yang et al., 2020b). LNPs, similar to those used in the COVID-19 mRNA vaccines (mRNA-1273 and BNT162b2), are also utilized for delivering circRNA vaccines and therapeutics (Wesselhoeft et al., 2019; Qu et al., 2022).

Mature mRNAs are well known for their crucial role in protein synthesis and their usefulness as transporters of genetic information to the location of the target cells. Recent research on severe acute respiratory syndrome coronavirus has found that exosomes loaded with lung mRNA encoding the spike protein of the virus demonstrated higher immune responses compared to liposomal counterparts. Notably, the exosomes remained stable even at room temperature and exhibited better *in vivo* distribution (Popowski et al., 2022).

LncRNAs possess a wide range of functional activities, presenting multiple avenues for therapeutic intervention, which necessitates tailoring the method of targeting to the specific mode of action of the lncRNA.A noteworthy advancement is the investigation of natural-antisense transcripts (NATs): lncRNAs transcribed in the reverse direction to coding genes, which suppress gene expression in a cis manner. Antisense oligonucleotides (ASOs) that target NATs, referred to as ‘antagoNATs’, have demonstrated remarkably promising preclinical outcomes for gene reactivation in the central nervous system (Modarresi et al., 2012). Likewise, exosomes can also be loaded with lncrnas, one article explores the regulation of exosomal lncRNA GAS5 on apoptosis of macrophages and vascular endothelial cells in atherosclerosis, which revealed that gas5 has potential therapeutic implications in the prevention of atherosclerosis (Chen et al., 2017b).

One of the primary advantages of CRISPR-Cas is its ability to target and edit DNA with high precision, enabling researchers to potentially cure genetic disorders by correcting the underlying genetic mutations responsible for the disease. At present, the CRISPR family has primarily utilized Cas9 and Cas12a for genome editing, which providing a valuable range of applications for CRISPR technology by utilizing just these two enzymes (Paul et al., 2020). Epithelial cell-derived microvesicles have been identified as safe and effective CRISPR/Cas9 carriers for treating cancer. These microvesicles demonstrate cancer cell tropism, leading to selective accumulation in tumors and exhibiting a synergistic anti-tumor effect (He et al., 2020).

Despite significant progress in the development of delivery systems for RNA therapeutics, including liposomes, polymers, and inorganic materials, delivering single RNA molecules remains a significant challenge for clinical applications. However, there is growing interest in the potential of exosomes as a superior delivery system, thanks to their low immunogenicity, excellent blood compatibility, and rapid delivery capabilities, making them an exciting area of ongoing research.

### Therapeutic potential of exosomes as co-delivery system for drug and RNA

The transition from exclusively loading extracellular vesicles with RNA and drugs to jointly loading them with both demonstrates the immense potential of extracellular vesicles as carriers. Previous studies have highlighted challenges associated with separate loading of RNA and drugs, including limited transportation capacity and delivery instability. However, thanks to extensive scientific research and technological advancements, simultaneous co-loading of RNA and drugs into extracellular vesicles has emerged as a new Frontier in the field.

Currently, various cancer treatment options are available, including peer therapy, chemotherapy, hyperthermia, immunotherapy, photodynamic therapy, radiation therapy, stem cell transplantation, surgery, and targeted therapy. However, single chemotherapy drugs only target a limited range of molecules and pathways, leaving some unexpected mutations unaddressed. In this regard, non-extracellular co-delivery systems have been previously reported (Chung et al., 2021). Studies have investigated the potential of using RNAs and anticancer drugs together to enhance the efficacy of cancer treatment. Yin et al. employed multifunctional magnetic core-shell nanoparticles for simultaneous delivery of microRNA therapeutics and anticancer drugs, while Xu et al. used liposomes to co-deliver miR-101 and doxorubicin and Zhu and others developed matrix metalloproteinase 2-sensitive multifunctional polymeric micelles for tumor-specific co-delivery of siRNA and hydrophobic drugs (Zhu et al., 2014; Xu et al., 2017; Yin et al., 2018). The results of these studies showcase the potential of combining RNA and anticancer drug therapies in order to overcome chemoresistance, and provide a hopeful strategy for improving cancer treatment. Nevertheless, the utilization of this approach for combined therapeutics in a single nanocarrier is currently confined to preclinical research, mainly due to issues related to manufacturing and regulation (Eljack et al., 2022).

With the most well studied nanocarriers being mesoporous silica nanoparticles, dendrimers, polymers, liposomes, and gold nanoparticles, exosomes as carriers for co-delivery systems still many techniques stay at the laboratory research level, while exosomes have unique advantages as endogenous natural drug carriers due to their biological membrane penetration, which can improve the transport efficiency and targeting of drugs, low degradation rate, and high permeation retention effect (Zhao and Feng, 2015; Pan et al., 2019; Si et al., 2020).

Patients with colorectal cancer often develop resistance to 5-fluorouracil (5-FU), which leads to poor treatment outcomes. MiR-21 has been identified as a marker for human malignant tumors and is also associated with multiple drug resistance (Shi et al., 2010; Moridikia et al., 2018). In this study, engineered exosomes targeting HER2 were obtained by constructing HEK293 cells overexpressing HER2 antibody, and 5-FU and miR-21 inhibitor were loaded into the exosomes using electroporation and targeted to HER2-positive tumor cells. The results demonstrated that engineered exosomes effectively promoted the uptake of 5-FU-resistant HCT-116 cells and significantly downregulated miR-21 expression, leading to cell cycle arrest, reduced tumor proliferation, increased cell apoptosis, and rescued PTEN and hMSH2 expression. Furthermore, systemic administration of these exosomes significantly reduced tumor growth in a mouse model (Liang G. et al., 2020b).

**FIGURE 2:**
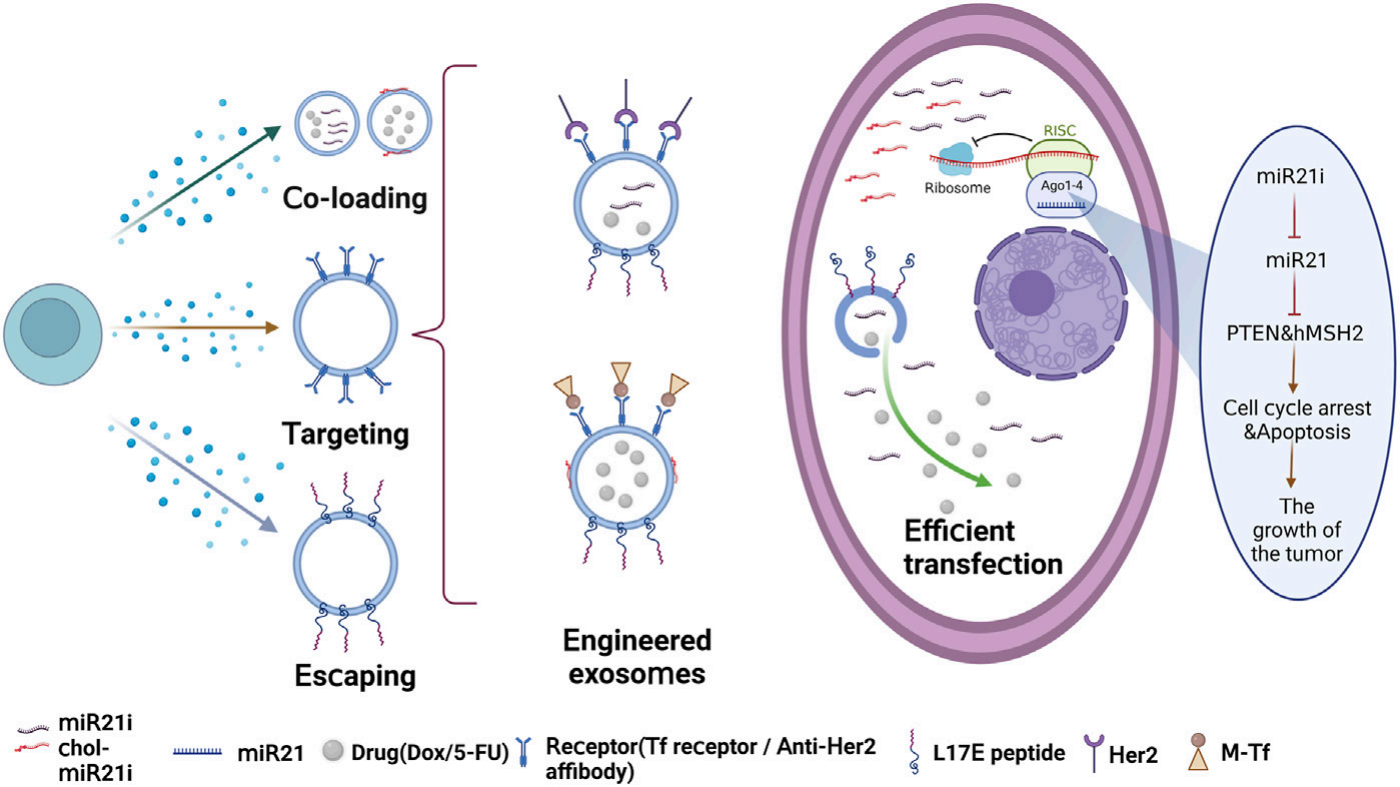
Effective antitumor therapy utilizing engineered exosomes with miR21i/chemo combination.

In a recent study, a blood exosome-based superparamagnetic nanoparticle cluster was constructed that incorporates tumor-targeting functions into exosomes. The researchers then assembled chemotherapeutic drug doxorubicin (Dox) and cholesterol-modified single-stranded miRNA21 inhibitor (chol-miR21i) onto the exosome to create a nanoplatform that combines two different anticancer modalities. To ensure efficient release of the cargos, including RNAs, a cationic lipid-sensitive endosomolytic peptide known as L17E peptide was introduced into this exosome-based co-delivery system. By combining both drugs and RNAs, this dual therapy interferes with nuclear DNA activity and downregulates the expression of oncogenes, leading to a significant inhibition of tumor growth and fewer side effects. Overall, this study shows the potential for exosome-based therapies to deliver multiple treatment modalities for more effective cancer treatment (Zhan et al., 2020). And Figure 2 shows the effective antitumor therapy utilizing engineered exosomes with miR21i/chemo combination.

In addition, the research team also tested the use of exosomes as delivery vehicles for RNA and small molecule drugs in treating glioblastoma (GBM). They engineered the exosomes to co-load sicpla2 and metformin in an efficient manner. The resultant exos met/sicpla2 construct was shown to significantly inhibit mitochondrial energy metabolism and ATP production in GBM. The study also demonstrated that systemic administration of exos met/sicpla2 was capable of efficiently penetrating the blood-brain barrier (BBB) and accumulating specifically in GBM in patient-derived xenograft models (Zhan et al., 2022).

**FIGURE 3:**
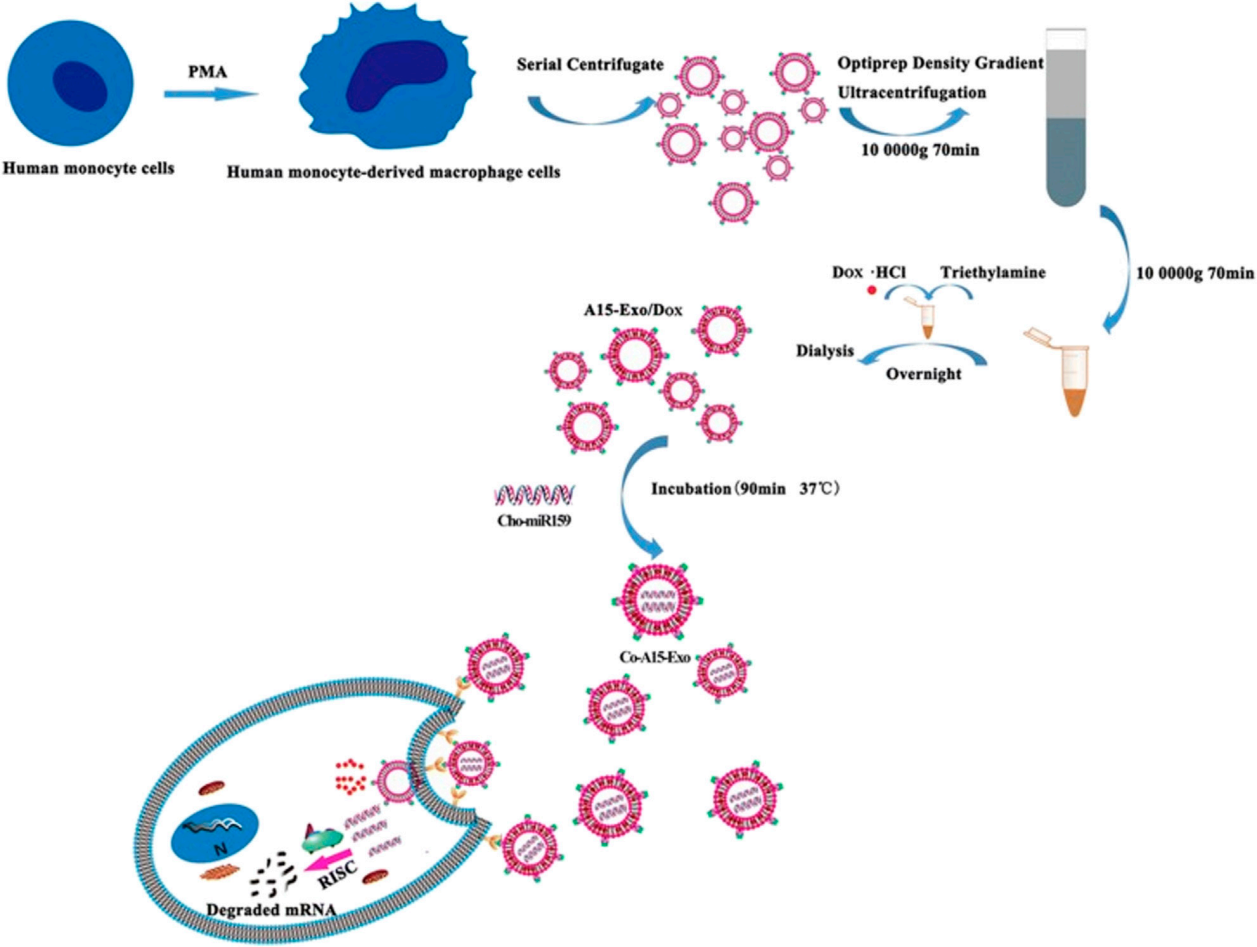
Formation and release of Dox and Cho-miR159-loaded A15-Exo (Co-A15-Exo) (Gong et al., 2019).

Exosomes there are limitations in their ability to specifically target certain recipient cell types. To address this issue, we have devised a strategy to isolate exosomes that exhibit increased binding to integrin αvβ3. This binding is facilitated by a modified version of a disintegrin and metalloproteinase 15 (A15) expressed on the exosomal membranes (A15-Exo). The A15-Exo were derived from monocyte-derived macrophages that were continuously activated by protein kinase C, resulting in cell-derived Exo exhibiting targeting properties and a 2.97-fold higher production yield. *In vitro* studies demonstrated that A15-Exo, co-loaded with Dox and Cho-miR159, exerted strong synergistic therapeutic effects on MDA-MB-231 cells. *In vivo*, vesicular delivery of miR159 and Dox effectively silenced the TCF-7 gene and exhibited improved anticancer effects without any adverse effects (Figure 3) (Gong et al., 2019).

The combination of small molecule drugs with other therapeutic modalities that utilize unique mechanisms of action is emerging as a promising approach for overcoming drug-resistant cancers. Nucleic acids are often incorporated into such strategies due to their highly specific targeting capabilities. In order to facilitate the simultaneous delivery of drugs and nucleic acids, researchers have developed and refined smart nanocarriers that preserve the physicochemical characteristics and biological functions of both components. More importantly, successful integration of exosomes has shown promise for reversing chemoresistance.

## The bottleneck of exosomes as carriers for delivery systems

Recent report has expressed doubts about the effectiveness of exosomes as a universal delivery system for RNAs, suggesting that anionic liposomes may perform better in this regard (Stremersch et al., 2016). Undoubtedly several challenges remain before meaningful clinical translation can be achieved, for example,

What are the standards for isolating, purifying, and quantifying exosomes? How should donor cells be selected, and what is the ideal cell culture environment to enable large-scale, industrial production of exosomes? While mesenchymal stem cells may be the first choice, are red blood cells, which are gene-free donor cells, the future stars?

Given that there is heterogeneity among different exosomes, how should exosomes be selected for drug or RNA loading methods? Will modifying exosomes affect their function and properties? After large-scale production of exosomes, what is the optimal long-term preservation method?

How do exosomes interact with the human internal environment, and what is their pharmacokinetic profile in body fluids?

The engineered production of exosomes involves multiple steps. Have quality control measures been put in place, and are personnel trained to ensure standardization?

Considering the aforementioned factors, it is important to address the challenges associated with co-loading RNA and drugs into exosomes. In addition to the factors mentioned earlier, achieving optimal loading capacity requires considering the appropriate ratio of drugs to RNA within the exosomes. Furthermore, it is essential to match the pH conditions that would result in the maximum release capacity for both the drugs and RNA within the exosomes. As mentioned in the literature, the researcher conducted a series of tests involving gradient concentrations of RNA and drugs, and explored different pH values in order to estimate the maximum release amount (Gong et al., 2019).

Before exosomes can be used for simultaneous RNA and drug delivery, several key issues must be addressed. A key issue is the identification and development of the optimal combination of RNA drugs for loading into extracellular vesicles, which not only requires specific targeting of disease sites, but also optimization of RNA drug ratios. Another challenge is to optimize drug release and extracellular transport mechanisms to effectively deliver drugs to target tissues. Further research is needed to modify exosomes to improve pharmacokinetics, such as half-life, biological distribution, and clearance rate.

## Perspective and discussion

Over the last few decades, researchers have increasingly focused on exploring the potential of exosomes as drug delivery systems. One emerging trend in cancer treatment involves utilizing combination therapy of drugs and nucleic acids delivered through exosomes. The clinicaltrials.gov resource (https://clinicaltrials.gov/ct2/home) indicates a significant number of clinical trial registrations for exosomes, including trials investigating the loading of exosomes with small molecule drugs (NCT01294072) and RNA molecules (NCT03384433). Exosomes offer several advantages as carriers of RNA and drugs. Firstly, they are naturally occurring and can be obtained from multiple cell types, providing a wide variety of targeting options while also potentially reducing immunogenicity. Secondly, exosomes are highly effective at transporting different molecules, including RNA and small molecules, in a protected manner which increases their stability and half-life. Lastly, exosomes possess tissue targeting mechanisms which allow them to deliver drugs directly to affected tissues and organs.

In summary, exosomes provide the potential to simultaneously load RNA and drugs for targeted and effective drug delivery.
